# Supplementary material for: Activated Dendritic Cell Subsets Characterize Muscle of Inclusion Body Myositis Patients and Correlate with KLRG1+ and TBX21+ CD8+ T cells
Source: medRxiv. 2025 Jun 5:2025.06.04.25328910. Preprint. [Version 1] doi: 10.1101/2025.06.04.25328910 (PMC12155022; doi:10.1101/2025.06.04.25328910)

Supplementary Figure 1. UMAP clustering of all filtered cells from snRNA-seq. UMAP representations of original cell clusters generated by FindClusters function (A), and cells labeled by dataset they originated from (B).

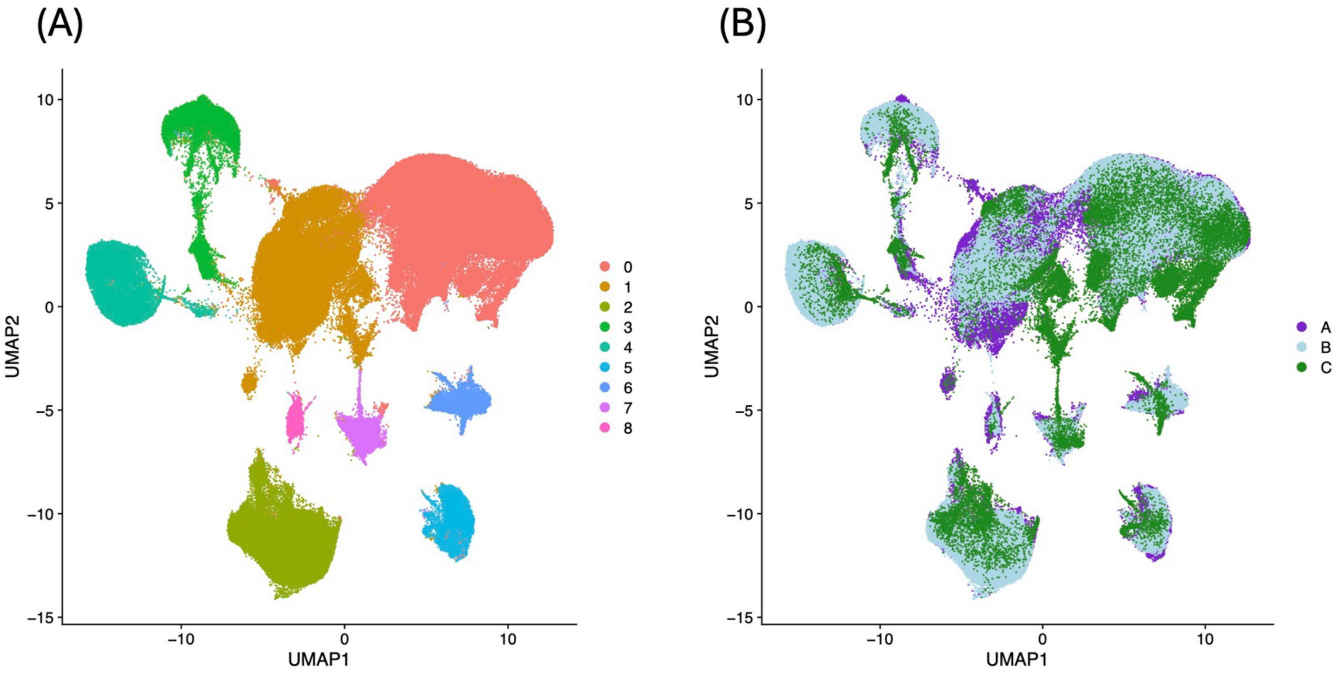

Supplementary Figure 2. FeaturePlot of representative genes for each cell cluster from snRNA-seq.

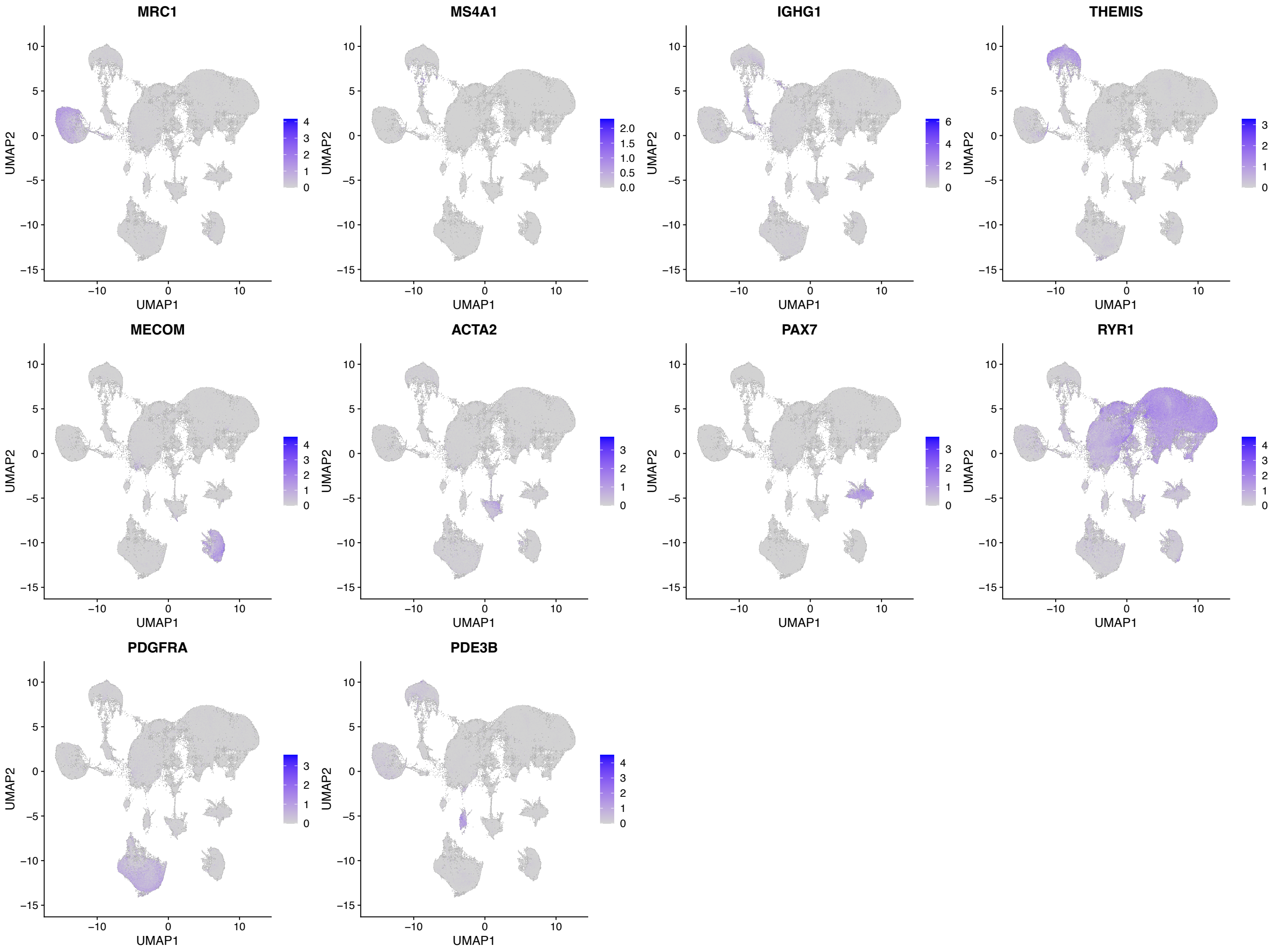

Supplementary Figure 3. UMAP clustering of Lymphocytes from snRNA-seq. UMAP representations of original cell clusters generated by FindClusters function (A), and corresponding named clusters (B).

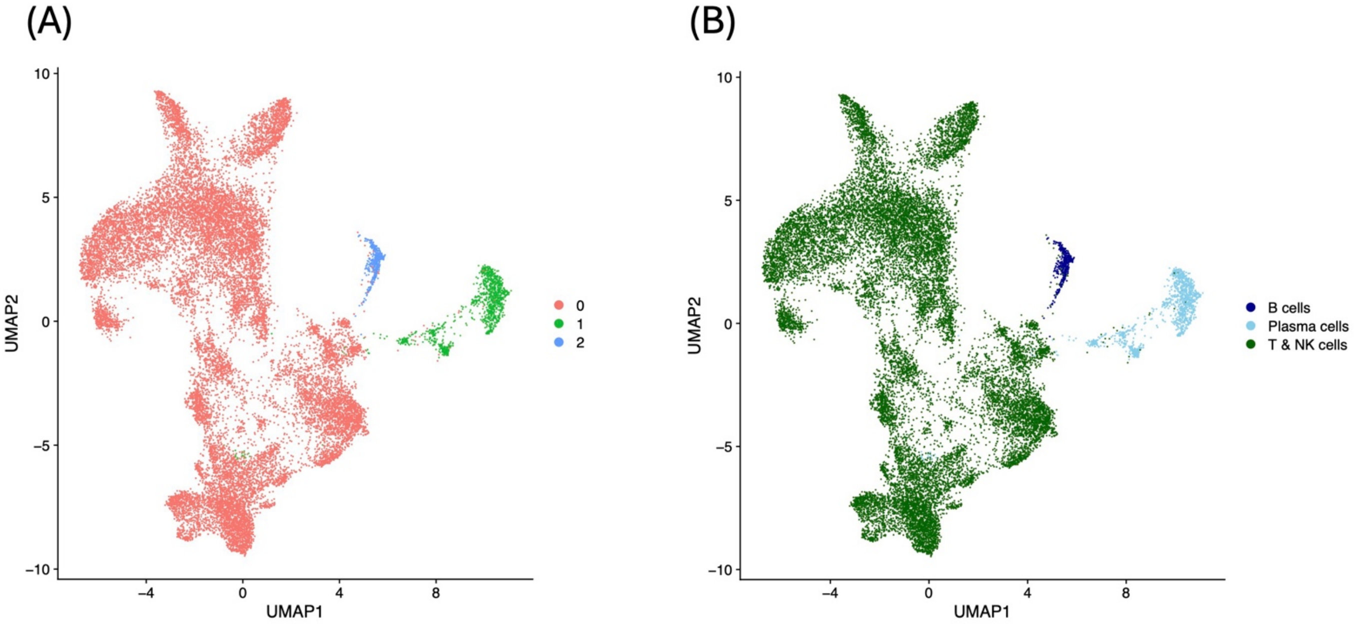

Supplementary Figure 4. FeaturePlot of lymphocyte gene expression for lymphocyte clusters from snRNA-seq. Showing expression of T cell markers (*THEMIS*, *ITGAE*, *CD3D*, *CD4*, *CD8A*, *FOXP3*), NK cell marker (*NCR1*), B cell markers (*MS4A1*, *PAX5*), and Plasma cell markers (*IGHG1*, *IGKC*, *PRDM1*).

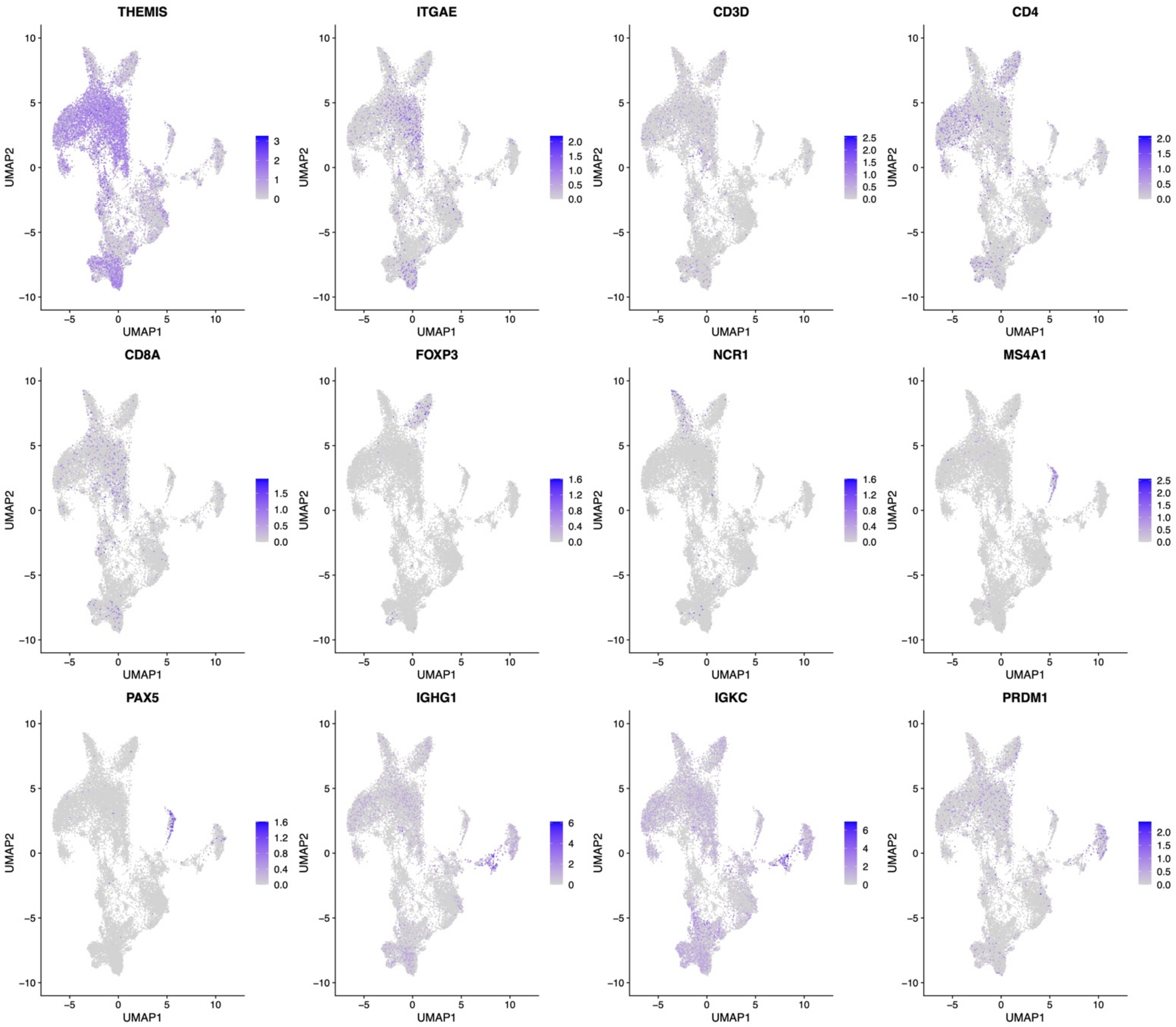

Supplementary Figure 5. UMAP clustering of Myeloid cells from snRNA-seq. UMAP representations of original cell clusters generated by FindClusters function (A), cells labeled by group (B), and cells labeled by dataset they originated from (C).

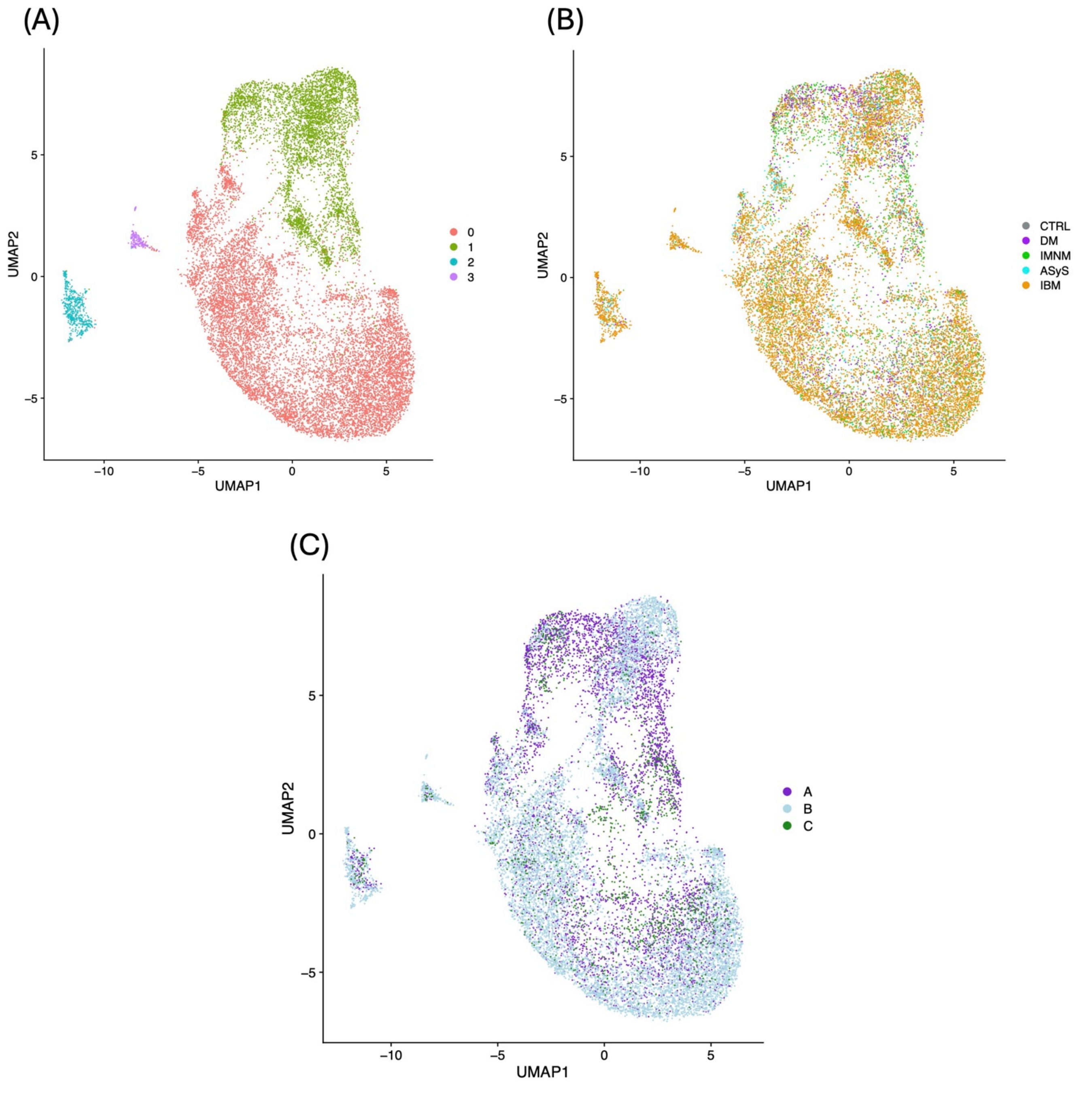

Supplement: Supplement 2 [file media-2.pdf]
